# Molecular mechanism of Alzheimer’s disease using integrated multi-omics

**DOI:** 10.3389/fnagi.2026.1735696

**Published:** 2026-03-13

**Authors:** Mera Alhusaini, Bashair M. Mussa, Burcu Yener Ilce, Hamid Alhaj, Rifat Hamoudi

**Affiliations:** 1 Research Institute for Medical and Health Sciences, Sharjah, United Arab Emirates; 2 College of Medicine, University of Sharjah, Sharjah, United Arab Emirates; 3 Center of Excellence for Precision Medicine, Research Institute of Medical and Health Sciences, University of Sharjah, Sharjah, United Arab Emirates; 4 BIMAI-Lab, Biomedically Informed Artificial Intelligence Laboratory, University of Sharjah, Sharjah, United Arab Emirates; 5 Division of Surgery and Interventional Science, University College London, London, United Kingdom

**Keywords:** Alzheimer’s disease, autophagy, genetic risk factors, metabolic dysfunction, multi-omics

## Abstract

Alzheimer’s disease (AD) is a devastating neurodegenerative disorder driven by complex interactions between neuroinflammation, immune dysregulation, metabolic impairment, and disrupted synaptic plasticity. Emerging evidence highlights maladaptive microglial activation, chronic cytokine signaling (including IL-1β, TNF-*α*, and IL-6), and hypothalamic–pituitary–adrenal (HPA) axis hyperactivity as pivotal contributors to neuronal damage and cognitive decline. Genetic studies further underscore the importance of immune and metabolic pathways, implicating key risk genes such as APOE, TREM2, and CR1, while deficits in autophagy exacerbate pathological protein aggregation, including amyloid-β and tau, ultimately accelerating synaptic loss. In this review, we synthesize molecular, genetic, and cellular evidence to clarify the mechanisms driving AD pathogenesis. We discuss genome-wide association study (GWAS) findings that define the genetic architecture of the disease, the neuroimmune crosstalk affecting memory-related brain regions, the link between chronic stress and amyloid pathology through HPA-axis dysregulation, and metabolic reprogramming in neurons, astrocytes, and microglia. Together, these interconnected processes highlight how dysregulated immunity and impaired protein clearance contribute to neuronal dysfunction and the progressive cognitive decline characteristic of AD.

## Graphical abstract

**Figure:**
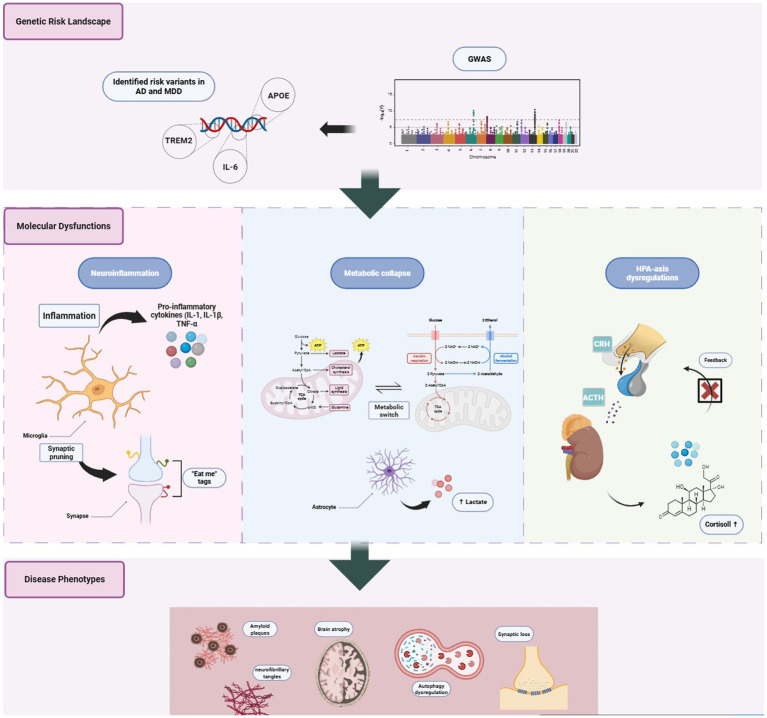

## 1 Introduction

Alzheimer’s disease (AD) is a devastating neurodegenerative disorder that primarily affects the elderly population. Recent data estimates a striking 416 million individuals worldwide with the AD continuum (prodromal, preclinical, and full AD dementia) (Alzheimer’s Association, 2017). Two-thirds of AD cases in the USA are women (Gustavsson et al., 2023) and AD is considered the fifth leading cause of death among women compared to being the eighth among men. Global epidemiological studies demonstrate similar trends. For example, in the Middle East and North Africa (MENA) region in 2019, the age-adjusted point prevalence of dementia was 777.6 cases per 100,000 people, being higher for women in all age groups (Safiri et al., 2023).

AD is characterized by progressive cognitive decline, including memory impairment and language difficulties, eventually leading to a significant functional impairment. One of the key neuropathological features of AD is the accumulation of amyloid-beta (Aβ) plaques and tau protein in the brain accompanied by widespread cellular alterations, including changes such as neuronal dystrophy, neuronal loss, astrogliosis, and microgliosis. Indeed, abnormally folded Aβ and hyperphosphorylated tau protein remained the core pathological hallmark for AD, backed by years of evidence supporting the casual relation to neurodegeneration. However, the disease’s complexity suggests a multi-factor pathology (Small and Duff, 2008) as Alzheimer’s involves protein pathology and is often linked to age-related conditions like cerebrovascular and Lewy body diseases, leaving its primary cause an active research area (Attems and Jellinger, 2014).

Immune response and neuroinflammation in particular have gained increased attention as a central component of the disease. In AD neuroinflammation seems to play a role in the pathology cascade that is more than a mere reaction to senile plagues and neurofibrillary tangles (Heneka et al., 2015). In a comprehensive review, Weaver identifies 30 diverse risk factors for Alzheimer’s disease (AD) and highlights neuroinflammation as the unifying mechanism underlying all these factors (Wang et al., 2023). The author argues that despite the varied nature of these risk factors, they all converge on the activation of microglia and the release of pro-inflammatory cytokines, such as IL-1β, IL-6, and TNFα. This neuroinflammatory response is implicated in neuronal loss and brain atrophy, contributing to the pathogenesis of AD.

This notion is asserted with the evidence of genes related to immune process and inflammations such as *TREM2*, *CD33, and CR1* are associated with AD (Katsel and Haroutunian, 2019; Li et al., 2023). Mutations in the gene coding for TREM2 (Triggering receptor expressed on myeloid cells 2) is a risk factor for AD (*p* value = 3.00E-37 risk allele: rs75932628-T) with odds ratio similar to that of APE4 (Jonsson et al., 2013; Kunkle et al., 2019). TREM2 is a transmembrane receptor that is almost exclusively expressed in microglia and regulates a wide range of functions in myeloid cells such as cell survival, proliferation and differentiation, and phagocytic processes (Jay et al., 2017).

Transcriptome and proteome analysis of human *APP*-KI mouse crossed with TREM2-KO, revealed that TREM2 acts as a master regulator of microglial immune response (Lin et al., 2024). Absence of TREM2 resulted in downregulation of genes related to antigen presenting such as IBA-1 and CD74 and lysosomal formation Ctsd and CD68 compared to APP-KI. This may be attributed to the reduction in the number of plaque-associated microglia. On the protein level, components of the complement system such as C1qa, C1qb, and C1qc were downregulated, these findings suggest that TREM2 deficiency disrupts microglial immune surveillance and response to amyloid pathology, potentially exacerbating neurodegeneration in AD.

Disruptions in autophagy and metabolic regulation are also pathological events in AD. Autophagy, the process responsible for clearing damaged cells and proteins, is impaired in AD, contributing to the accumulation of toxic proteins (Gassen and Rein, 2019; Zhang et al., 2021). This complexity presents a compelling avenue for research, potentially holding the key to understanding the multifactorial nature of AD. In this review, we aim to examine the major theories and recent evidence underlying AD pathogenesis, with a particular focus on genetic susceptibility, immune system involvement, and metabolic alterations that converge to drive disease progression.

## 2 Genetic susceptibility

### 2.1 Molecular mechanisms of major genetic determinants in Alzheimer’s disease

Based on the age of onset of the first symptoms, AD can be categorized as Early (also known as familial) and Late (also known as sporadic) -onset AD (EOAD and LAOD), where EOAD affects individuals under the age of 65 and accounts for 1–5% of all AD cases (Dorszewska et al., 2016). In contrast, LOAD effects population of ages above 65 years of age (Mendez, 2017). The two subtypes present differences beyond the onset of symptoms, including clinical manifestations, neuropathology and neuropsychological profiles. It has been reported that the main genetic causes of EOAD are identified as mutations in genes such *APP* on chromosome 21, presenilin 1 (*PSEN1*) on chromosome 14, and presenilin 2 (*PSEN2*) on chromosome 1 (Levy-Lahad et al., 1995).

#### 2.1.1 Early onset Alzheimer’s disease

Amyloid Precursor Protein (APP) is a type 1 membrane protein, expressed widely in the body, with higher expression in the brain, specifically excitatory neurons and GABAergic (gamma-aminobutyric acid) interneurons, with higher levels being in the cortex and hippocampus (Hick et al., 2015; Wang et al., 2014). APP has a wide function profile; it plays a cell-intrinsic role in regulating synaptic plasticity and memory in mature excitatory neurons of the cerebral cortex (Arancio, 2020). This is attributed to changes occurring in both pre- and post-synaptic compartments. Additionally, APP is involved in controlling synaptic transmission, plasticity, and maintaining calcium balance within neurons. Its functions extend to developmental stages and include exerting neuroprotective effects (Arancio, 2020; Hefter et al., 2020). Mice models lacking *APP* present long-term memory deficits and age-dependent dysfunctions in passive avoidance acquisition (Senechal et al., 2008).

Aβs, which largely consist of peptides that are 40 and 42 amino acids long (Aβ40 and Aβ42), are produced by sequential APP proteolytic processes by beta-site APP-cleaving enzyme (BACE) and *γ*-secretase.

In the amyloidogenic pathway, initially, APP undergoes cleavage by β-secretase at the amino terminus of Aβ, resulting in the release of a soluble form of APP (sAPPβ) and the formation of membrane-bound C99. Additionally, proteolytic cleavages of C99 by *γ*-secretase lead to the release of Aβs and the intracellular domain of APP.

Mutations in the APP gene can cause a shift in its localization from the cell surface to early endosomes, this dysregulated trafficking of APP and BACE to the same subcellular compartments can enhance their interaction, promoting the amyloidogenic pathway and excessive production of Aβ peptides. This aberrant trafficking may lead to Aβ accumulation, culminating in the formation of neuritic plaques. Disturbances in APP trafficking affecting its internalization and processing in endosomes/lysosomes can tip the balance towards amyloidogenic pathways, favoring Aβ production (Zhang and Song, 2013; Li et al., 2019).

*PSEN1* and *PSEN2* codes for two transmembrane proteins of the four proteins in *γ*-secretase. They are involved in the production of Aβ42 through the C-terminal membrane region of APP. While PSEN1 and PSEN2 are not enzymes by themselves, they play a crucial role in regulating *γ*-secretase cleavage, PSEN1 can also play a role in the transport of C-terminal APP fragment to the γ-secretase complex Similarly, PSEN2 may induce γ-secretase activity (Cai et al., 2015; Bagaria et al., 2022).

#### 2.1.2 Late onset Alzheimer’s disease

Despite numerous extensive GWAS and GWAS meta-analyses, the ε4 variant of the apolipoprotein gene (*APOE*) remains the most potent genetic risk factor linked to LOAD. There are three primary isoforms of the *APOE* gene, referred to as ε2, ε3, and ε4. ε4 impact increases in a gene dose-dependent manner, potentially elevating the risk by up to 15 times in homozygotes (Strittmatter et al., 1993a). Conversely, *APOE2* reduces the risk of AD by nearly half and is linked to increased longevity (Corder et al., 1994).

In AD, APOE4 (the protein coded by ε4 variant of *APOE*) was shown to play a role in the initiation and progression of the disease through a wide range of pathways, disputing the previous believe that restricted the APOE role in triggering Aβ accumulation (Blumenfeld et al., 2024). In the periphery, APOE is mainly produced by hepatocytes and macrophages in the liver (Huang et al., 2004), however, in the CNS, APOE main source is activated microglia, astrocytes, choroid plexus cells, and vascular mural cells (Pitas et al., 1987; Xu et al., 2006). APOE acts as a lipid and cholesterol transporter, binding to neuronal low density lipoprotein receptors to transport lipids into neurons, this activity is mainly mediated by members of the low-density lipoprotein receptors (LDLR) including very low-density lipoprotein receptor (VLDLR), the APOE receptor 2 (APOER2), and the LDL receptor-related protein (LRP).

The LDLR family is likely to mediate the clearance of Aβ through receptor mediated clearance. APOE is thought to facilitate binding of Aβ to these receptors, APOE binds to Aβ through its C-terminal domain similar to lipid binding (Tamamizu-Kato et al., 2008; Strittmatter et al., 1993b). ApoE3 binds to Aβ with higher affinity than APOE4, leading to a better clearance of Aβ (LaDu et al., 1994), which was confirmed by human brain imagining that showed greater Aβ burden in patients with the *APOE4* allele (Baek et al., 2020; Mishra et al., 2018). Moreover, studies have shown that Aβ bound to APOE4 is cleared through VLDLR rather than LRP1 and VLDLR, which is associated with slower internalization and clearance of the complex (Deane et al., 2008).

Notably, APOE3/lipoproteins have been reported to exert a protective role against apoptosis as appose to APOE4’. Phosphorylation of Ser-9 on glycogen synthase kinase-3β (GSK3) decreases during apoptosis, but this reduction is reversed by the addition of lipoproteins. This indicates that these lipoproteins potentially safeguard neurons from apoptosis by deactivating (GSK3) through direct or indirect phosphorylation at Ser-9 by protein kinase Cδ (PKCδ). Lipoproteins that contain APOE4 were found significantly less protective (Hayashi et al., 2007). APOE interacts with transmembrane ATP-binding cassette (ABC) transporters such as ABCA1 and ABCG1to allow loading of lipids, making its role critical for synapse formation and tissue repair (Bu, 2009). Amino acid substitutions between ε4 and ε2, ε3 leads to major alternations in functionality, disrupting lipid and receptor binding, and stability (Ruiz et al., 2005; Hatters et al., 2006).

Several studies demonstrated other functions of APOE in the CNS, APOE was found to effect neurite outgrowth in an isoform-specific manner; *APOE* knock-out mice showed diminished neurite outgrowth compared to wild type (Nathan et al., 2002), this was also observed in embryonic hippocampal neurons cultured on a monolayer of astrocytes obtained from mice lacking *APOE* (Narita et al., 1997). Moreover, conflicting impacts of APOE3 and APOE4 were observed on neurite outgrowth in adult neurons; APOE3 promoted while APOE4 hindered this process (Hussain et al., 2013). The effect was shown to be mediated by LRP; inhabiting the function of LRP neutralized the effect of both isoforms on neurite outgrowth. This isoform-dependent difference in the exerted effect may be attributed to the higher accumulation of APOE3 in the neurites compared to apoe4, which may be associated with microtubule depolymerization as neurons treated with apoe4 showed fewer microtubules and reduced polymerized to monomeric tubulin ration than apoe3 (Nathan et al., 1995).

Although a complete understanding of the mechanism in which APOE effects neurite outgrowth is still lacking, an association with microtubule formation have been demonstrated in numerous studies. Cultured neuronal cells treated with aoE4 exhibited a decreased number of microtubules and a significantly lower ratio of polymerized to monomeric tubulin compared to cells treated with APOE3 (Nathan et al., 1995). The diminished ability of APOE4 to interact with microtubule associated proteins such as tau and microtubule-associated protein 2c (MAP2c) shown by biochemical studies, reduces their ability to interact and stabilize microtubules (Huang et al., 1994).

ApoE4 has been reported to play a major role in the cognitive dysfunction seen in AD. For instance, both transgenic mice expressing APOE4 and mice with APOE4 knock-in display learning and memory deficits that are dependent on age and sex, even in the absence of Aβ accumulation, in contrast to transgenic mice expressing APOE3 and mice with APOE3 knock-in (Leung et al., 2012). These dysfunctions may be mediated by the effects of APOE4 on synaptic plasticity, mainly due to APOE4 role in neurotransmitter receptor expression and recycling. APOE4 has been found to specifically hinder synaptic plasticity and N-methyl-d-aspartate (NMDA) receptor phosphorylation by Reelin; a crucial regulator of brain development and synaptic strength modulation. ApoE4 was also shown to reduce the expression NMDA and *α*-amino-3-hydroxy-5-methyl-4-isoxazolepropionic acid (AMPA) receptors, this significantly diminishes Reelin’s capacity to enhance synaptic glutamate receptor function (Chen et al., 2010).

Inflammation in AD has emerged as a possible link between different pathological hallmarks in AD including Aβ aggregates, tau hyperphosphorylation, and even cognitive impairments (Kinney et al., 2018). While inflammation is a physiological reaction to infection or toxins in the brain, an uncontrolled response or imbalance between pro- and anti-inflammatory regulators can lead to sustained harmful neuroinflammation, that presents as microglial activation and proinflammatory cytokines release. This is widely seen in postmortem tissues of AD patients (Hopperton et al., 2018; Rao et al., 2011) and preclinical models (Verheijen et al., 2022; El-Nashar et al., 2022). the role of APOE in inflammation has recently gained much attention, one study compared a panel of inflammatory related genes between APOE3/E3, APOE4/4 carriers with AD pathology and APOE3/3 control, the results showed E4 carriers to exhibit an altered inflammatory response compared to E3 carriers, particularly in genes associated with microglial activation. This suggests a dampened immune response in AD patients carrying the E4 allele, resembling the restrained microglial activity observed in E3 carriers without AD pathology. One interesting finding is related to spalt like transcription factor 1 (SALL1); this transcriptional factor was found to be a master regulator of the microglia in its non-reactive state and allows for their function in hemostatic conditions in the brain (Buttgereit et al., 2016). Proteins related to inflammation and activation were among the top 50 genes with altered expression in SALL1-deficient microglia isolated from mice. Downregulation of SALL1 in the presence of neurodegeneration allows for the transition of microglia from resting to disease-related microglia (Deczkowska et al., 2018). In E3 carriers SALL1 was downregulated, however no change was noted between E4 carriers and disease-free E3 controls. This may suggest that microglia carrying E4 may be unable to switch from a resting phenotype which hinders its ability to clear Aβ in early stages of the disease.

### 2.2 GWAS-identified risk loci and multi-omics insights into Alzheimer’s disease susceptibility

Risk alleles are genetic variants that increase susceptibility to developing certain diseases. In the context of AD, identifying these alleles is crucial for understanding the underlying genetic mechanisms that contribute to the pathophysiology of this disorder.

To identify risk alleles associated with AD, a comprehensive search was conducted in the GWAS Catalog^1^, a curated resource of GWAS findings. This research focused on genetic variants linked to AD (Table 1), extracting relevant alleles associated with disease risk. The identified risk alleles were then sorted by their *p*-values to prioritize those with the strongest statistical significance. From this analysis, the top 10 genes with the lowest *p*-values were selected, representing the most robust genetic associations.

**Table 1:** Top 10 genetic loci associated with AD risk based on GWAS data.

Risk allele	Mapped genes	Trait name	*P* value	OR value	mRNA expression	Refs.
rs438811-T	APOE, APOC1	AD (MTAG)	0.00E+00	-	↑ (TC)	Yamada et al. (1995)
rs429358-?	APOE	AD	2.00E-303	3.21		
rs429358-?	APOE	LOAD	1.00E-300	-		
rs146275714-?	NECTIN2	AD or family history of AD	1.00E-300	-	-	-
rs429358-?	APOE	AD or family history of AD	1.00E-300	-	↑ (TC)	Yamada et al. (1995)
rs12691088-?	APOC1	AD or family history of AD	1.00E-300	-		
rs2075650-G	TOMM40	AD	1.00E-295	2.53	↓ (PB) ↑ (MFG)	Lee et al. (2012) Lee et al. (2021)
rs75627662-T	APOC1, APOE	AD (MTAG)	7.00E-292	-	↑ (TC)	Yamada et al. (1995)
rs429358-C	APOE	AD (onset between ages 58 and 79)	5.00E-286	3.93		
rs41289512-G	NECTIN2	AD or family history of AD	6.00E-276	-	-	-

OR, odds ratio; MTAG, Multi-Trait Analysis of GWAS. Temporal cortex TC; peripheral blood PB; middle frontal gyrus MFG.

Among the top genetic loci associated with AD, the *APOE* gene stands out as the most significant and well-established risk factor, particularly the ε4 allele, which markedly increases susceptibility to late-onset AD. APOE plays a critical role in lipid metabolism, amyloid-β aggregation, and neuroinflammatory processes as discussed earlier. Recent studies highlight that its effect may be modulated by neighboring variants, including *TOMM40* and *APOC1*. Polygenic profiles combining these variants have shown distinct associations with cerebrospinal fluid (CSF) and plasma biomarkers, particularly Aβ42 and tau (Kulminski et al., 2022).

These nearby genes are frequently co-inherited with APOE due to their close genomic proximity and may contribute independently to AD risk. *APOC1* has been implicated in modulating inflammatory responses and cholesterol transport, while *TOMM40* is involved in mitochondrial protein import, with certain polymorphisms influencing the age of AD onset and promoting neuroinflammatory pathways (Kulminski et al., 2022; Zhang et al., 2024; Chen et al., 2023).

Although the APOE gene cluster is often described as a tightly linked genomic region, emerging transcriptomic evidence suggests a more nuanced regulatory architecture within this locus. Integrative analyses of postmortem human brains have demonstrated that while APOE and APOC1 mRNA levels closely track overall cluster expression, TOMM40 exhibits distinct age- and disease-dependent transcriptional behavior, with reduced expression in younger AD cases independent of global APOE cluster patterns (Haghani et al., n.d.). Proteome-wide association studies integrating AD GWAS data have demonstrated that transcriptomic and proteomic associations show only partial concordance, with aligned mRNA and protein-level effects observed for a limited subset of loci (Wingo et al., 2021). Notably, the genes discussed here do not fall within this concordant subset identified in large-scale ROSMAP-based analyses, highlighting the importance of post-transcriptional regulation and multi-layered molecular control in AD (Wingo et al., 2021).

Taken together, these findings suggest that the chromosome 19 risk locus is regulated in a more complex manner than often assumed, with shared genetic risk existing alongside gene-specific transcriptional and proteomic effects. These patterns highlight the value of multi-omics approaches for understanding how closely linked genes contribute to AD.

Another gene within the chromosome 19 risk region, NECTIN2 (PVRL2), has emerged as an independent contributor to Alzheimer’s disease susceptibility. Genetic association studies have consistently implicated variants at this locus, particularly rs6859, in AD risk (Kunkle et al., 2019; Lambert et al., 2013). Recent mediation analyses further suggest that the effect of rs6859 on disease risk is partially mediated through elevated cerebrospinal fluid pTau-181 levels, linking NECTIN2 variation to tau-related neurodegenerative processes (Rajendrakumar et al., 2024a). In longitudinal analyses, NECTIN2 variants have been associated with differential trajectories of cognitive decline, suggesting that genetic variation at this locus may contribute to inter-individual heterogeneity in disease progression (Rajendrakumar et al., 2024b). Functionally, NECTIN2 encodes a cell-adhesion molecule of the nectin family that plays roles in synaptic organization, intercellular junctions, and immune-cell interactions (Liu et al., 2012). Multi-omic analyses provide additional support for its biological relevance; the NECTIN2 locus exhibits both cis-eQTL effects in human brain tissue and cis-pQTL effects in cerebrospinal fluid, indicating that genetic variation influences both mRNA expression and protein abundance (Wang et al., 2024). Notably, functional validation experiments demonstrate that NECTIN2 overexpression modulates soluble TREM2 levels, suggesting a potential link between this locus and microglial signaling pathways implicated in AD (Wang et al., 2024). Together, these findings indicate that NECTIN2 contributes to Alzheimer’s disease through mechanisms that extend beyond simple linkage with APOE, involving coordinated genetic, transcriptional, and proteomic regulation.

The observation that several of the most significant AD-associated variants converge on genes involved in immune regulation is important to highlight. Importantly, large-scale systems-level analyses have demonstrated that these immune and lipid-associated risk loci do not operate as isolated signals but are embedded within coordinated molecular networks. Network-based integrative analyses integrating genomic and transcriptomic data across multiple brain cohorts have identified AD-associated gene modules enriched for immune response, synaptic signaling, and metabolic pathways. For example, Wang et al. applied a systems biology framework to define co-expression modules that capture the convergence of genetic risk variants on microglial and inflammatory pathways, highlighting regulatory hubs rather than single-gene effects (Wang et al., 2018). Similarly, Wan et al. (2020) employed integrative network modeling approaches to delineate disease-associated transcriptional modules, revealing that AD susceptibility genes cluster within coordinated immune and glial regulatory networks. These findings reinforce the concept that AD pathogenesis reflects network-level dysregulation across molecular layers rather than parallel, independent biological processes (Wan et al., 2020). Notably, these network-level observations are consistent with large-scale GWAS meta-analyses, which have repeatedly identified immune and lipid-processing pathways as core components of the genetic architecture of Alzheimer’s disease. Large-scale GWAS meta-analyses have consistently demonstrated that pathways related to immunity and lipid processing represent central components of the genetic architecture of Alzheimer’s disease, rather than secondary consequences of neurodegeneration (Kunkle et al., 2019; Lambert et al., 2013). Importantly, integrative analyses across genetic and transcriptomic datasets indicate substantial inter-individual heterogeneity in AD, with distinct molecular subtypes characterized by differential immune activation signatures (De Jager et al., 2018). These findings suggest that immune-related loci may contribute to disease susceptibility and to variability in clinical manifestations and disease progression.

In this context, single-nucleus transcriptomic profiling of human prefrontal cortex demonstrates that substantial transcriptional remodeling occurs early in Alzheimer’s disease, prior to severe neurofibrillary pathology or advanced cognitive decline (Mathys et al., 2019). Notably, early gene-expression changes in glial populations are enriched for immune-related regulators. In microglia, genes such as APOE, C1QC, RASGEF1B, and CD83 are differentially expressed, consistent with activation of innate immune and complement pathways. In astrocytes, early upregulation of GFAP, SLC1A2, and CEMIP2 reflects reactive and metabolic reprogramming states (Mathys et al., 2019). These findings indicate that immune and glial transcriptional activation is detectable at early disease stages, supporting the view that neuroinflammatory processes are embedded in the initial molecular frame of Alzheimer’s disease.

Notably, emerging evidence indicates that *APOE* may interact with hypothalamic–pituitary–adrenal (HPA) axis dysfunction to accelerate AD pathogenesis. The APOE ε4 allele has been specifically linked to elevated CSF cortisol levels in both AD patients and non-demented older adults (Peavy et al., 2007; Peskind et al., 2001), suggesting a genotype-dependent alteration in HPA axis regulation. This genetic predisposition appears to create heightened vulnerability to environmental stressors, as demonstrated by studies showing that ε4 carriers experiencing prolonged stress exhibit significantly worse memory performance and higher cortisol concentrations than non-carriers under similar conditions (Peavy et al., 2007). The interaction between genetic risk and HPA axis dysfunction may be further modified by personality factors, with neuroticism and extraversion amplifying the adverse effects of APOE ε4 on cognitive outcomes (Dar-Nimrod et al., 2012).

## 3 HPA-axis

HPA-axis dysregulation can worsen memory loss. CSF cortisol levels were found to be associated with the worst disease progression and cognitive decline in patients with MCI and AD (Popp et al., 2015). High cortisol plasma levels were reported in AD early in the disease and preceding cognitive impairment (Hebda-Bauer et al., 2013; Ennis et al., 2017), and urine-free cortisol levels were found to predict AD risk on average of 2.9 years prior to onset (Ennis et al., 2017). Notably, several longitudinal analyses suggest that cortisol elevation may precede overt dementia, particularly in individuals with biomarker evidence of amyloid pathology (Holleman et al., 2022), indicating potential stage-dependent effects rather than uniform dysregulation across all patients. AD and MCI patients present higher levels of cortisol compared to healthy controls (Zheng et al., 2020; Dronse et al., 2023). Although these findings are broadly consistent across observational cohorts, variability in sampling methodology (plasma vs. CSF vs. urinary measures), circadian timing, and disease stage contributes to heterogeneity in reported effect sizes.

Higher cortisol levels were significantly associated with smaller left hippocampal volumes and, indirectly, with reduced memory function via reduced hippocampal volume. Additionally, elevated cortisol levels were related to reduced gray matter volume in the hippocampus and in temporal and parietal regions (Dronse et al., 2023). However, the magnitude of these associations varies across studies, and cortisol levels show substantial overlap between diagnostic groups, suggesting that while biologically relevant, cortisol alone lacks sufficient specificity as a diagnostic biomarker (Holleman et al., 2022). Together, these findings indicate that HPA-axis alterations are detectable early and associate with structural and cognitive vulnerability; however, whether cortisol elevation functions as an upstream driver, a downstream consequence of hippocampal degeneration, or part of a bidirectional feedback loop remains unresolved.

On the other hand, neurodegeneration of the hippocampus in AD (Mecca et al., 2022; Chiquita et al., 2019) creates a cycle that further exacerbates HPA-axis dysfunction. Under typical conditions, cortisol binds to GR in the pituitary, hippocampus, and paraventricular nucleus (PVN) to inhibit further secretion of corticotropin-releasing hormone (CRH). Neuronal degeneration in these areas impairs this negative control, thereby supporting sustained activation of the HPA axis.

However, the mechanisms by which this endocrine dysfunction translate into cognitive failure extend beyond neuronal toxicity alone. A critical pathway involves the dysregulation of microglia by chronic stress and elevated cortisol. Cortisol-driven hyperactivation of the HPA-axis primes microglia toward a pro-inflammatory state (Milligan Armstrong et al., 2021), disrupting their roles in synaptic maintenance and plasticity. Transcriptomic analyses of early AD brains consistently demonstrate upregulation of immune control genes in microglia, including complement components and APOE-associated inflammatory programs (Mathys et al., 2019; Olah et al., 2020). Given that glucocorticoids modulate microglial inflammatory responses in experimental models (Wohleb et al., 2018), it is plausible that stress-related endocrine signaling may intersect with these immune pathways and influence microglial state dynamics. Consequently, the interface between stress physiology and the brain’s immune system emerges as a crucial nexus for understanding cognitive decline (Calcia et al., 2016). This immune dimension suggests that HPA-axis dysfunction may modulate AD progression not only through neuronal damage but through altered glial activation states.

Glucocorticoids can directly boost BACE1 expression through both genomic and non-genomic mechanisms. GRs attach to the BACE1 promoter to increase transcription, while membrane-bound GRs activate CREB, which also binds to the BACE1 promoter (Green et al., 2006; Choi et al., 2017). Cortisol was found to induce Aβ in mitochondria prior to extracellular deposition by enhancing PSEN1 positioning in mitochondrial-associated membranes and inducing *γ*-secretase activity. Inhibiting GRs in corticosterone-treated mice reduced Aβ and improved spatial memory performance (Choi et al., 2023). Most mechanistic evidence derives from experimental models, and the relevance of physiological cortisol fluctuations in humans remains unclear.

Cortisol exerts its effects through GRs and MRs, widely expressed in learning and memory regions such as the hippocampus (Alhaj and McAllister-Williams, 2008; Alhaj et al., 2008). In AD, GR expression was decreased in the frontal cortex, while MR expression was elevated (Gil-Bea et al., 2010), resulting in a higher MR/GR ratio. MR expression negatively correlated with cognitive performance and positively correlated with Aβ levels. Interestingly, GR expression remained similar to synaptic markers, suggesting that reduced GR may reflect synaptic density loss rather than primary HPA dysfunction (Gil-Bea et al., 2010).

Collectively, the interplay between sustained cortisol exposure, impaired hippocampal feedback, altered receptor balance, and microglial activation supports a bidirectional model in which HPA-axis dysfunction both contributes to and is amplified by Alzheimer’s pathology. This framework highlights the need to consider endocrine–immune interactions within broader multi-omics analyses, rather than interpreting cortisol elevation as an isolated endocrine abnormality.

## 4 Brain immune system and cognition

Recent research has revealed that the immune response in the brain is not solely a defensive mechanism but also a key regulator of neural plasticity, memory formation, and mood (Yirmiya and Goshen, 2011). Starting during embryonic development, the brain resident immune cells-microglia- play a vital role in pruning synapses and neurogenesis, contributing to the adult cortical architecture (Paolicelli et al., 2011; Schafer et al., 2012). This intricate neuroimmune crosstalk continues into adulthood, where immune signaling molecules such as cytokines modulate synaptic function, plasticity, and memory as will be discussed below (Brioschi et al., 2020; Liu et al., 2021).

### 4.1 Neuroimmune crosstalk

Microglia play a direct and vital role in neuromodulation under physiological conditions, whether by physical contact or through multiple receptors and signaling pathways, as mentioned earlier, the complement system acts as “tags” for pruning weak or immature synapses and subsequent engulfing. Microglia not only prune synapses but also interact with engram neurons, the physical representations of memories in the brain, as they are the neurons that are involved in different steps of memory formation (encoding, consolidation, and retrieval) (Guskjolen and Cembrowski, 2023). The inhibition of the complement system using CD55 or depletion of microglia hindered the natural forgetting process and impaired the uncoupling of engram cell networks (Wang et al., 2020). These findings highlight how immune signaling, particularly cytokine production by microglia, contributes to cognitive processes, with IL-1β, TNF-*α*, and IL-6 standing out as key regulators.

IL-1β was reported to interfere with contextual fear memory formation (Gonzalez et al., 2013), an effect that might be mediated by activating P38/MAPK pathway in dorsal hippocampus. The intrahippocampal injection of IL-1β also delayed ERK phosphorylation and reduced BDNF expression and glutamate release.

TNF-*α* role in learning and memory was only reported in few studies. Mice lacking TNF-α preformed worse in novel object recognition task and Barnes maze than control (Baune et al., 2008). On the other hand, mice injected intraperitoneally with TNF-α had better performance in avoidance test (Brennan et al., 2004). TNF-α was also found to regulate synaptic plasticity, under physiological conditions, TNFα acts as a key permissive factor for glutamatergic gliotransmission in the hippocampal dentate gyrus. Although astrocytes can still exhibit calcium signaling in its absence, TNFα is required to enable the vesicular release of glutamate, which is essential for modulating synaptic activity (Santello et al., 2011).

IL-6 is a pleiotropic cytokine that plays a dual role in the central nervous system, contributing to both homeostatic and pathological processes. Under physiological conditions, IL-6 is involved in synaptic plasticity, neurodevelopment, and memory formation (Gruol, 2014; Balschun et al., 2004). However, its role is highly context-dependent, with both beneficial and detrimental effects reported depending on its concentration, timing, and cellular origin (Erta et al., 2012; Raison et al., 2006). In the healthy brain, low levels of IL-6 may support neuronal communication and neurogenesis, while sustained or excessive production, especially during neuroinflammation—has been linked to cognitive impairment and neurodegeneration (Marsland et al., 2008; Weaver et al., 2002). These findings underscore a complex, bidirectional role for immune signaling in the brain, balancing the mechanisms that underpin healthy cognitive function against those that may drive mood disorders.

In a chronic IL-6 overexpression model, working memory but not associative learning was compromised as evident by Y-maze and fear conditioning test, respectively, (Marguerite Wagnon et al., 2023). mRNA expression levels for a battery of markers were tested in different areas of the brain, interestingly, the cerebellum showed the greatest differences in transcriptomic profile. IBA-1, TREM2, and NF-ΚB levels were increased, which indicate microglial activation and inflammatory reaction. Moreover, the apoptotic marker Cas3 levels increased while the neuro-marker Ero2 decreased, this may indicate a loss on neuron numbers in the cerebellum.

### 4.2 Immune system as a mediator of experience

The brain is not solely influenced by external stimuli and experiences, but is also intricately modulated by the immune system, which responds dynamically to these experiences, This dynamic relationship between immune signaling and neural activity plays a crucial role in processes like neuroplasticity, memory formation, and cognitive function, demonstrating the immune system’s active involvement in shaping both brain structure and behavior.

Components of the innate immune systems such the transcription factor NF-κB—play dual roles in promoting synaptic plasticity and cognitive reserve, while also contributing to neuroinflammation and pathological conditions. NF-κB serves as a central mediator that converts synaptic activity into long-term changes in gene expression essential for memory formation (Oikawa et al., 2012; Albensi and Mattson, 2000). Upon neuronal stimulation—often triggered by synaptic events like calcium influx—NF-κB is activated and translocate from the synapse to the nucleus. There, it coordinates a transcriptional program that involves key genes such as JunD, MHC Class I (and II with beta 2 microglobulin), prodynorphin, BDNF, and the more recently identified IGF2 (Gobin et al., 2001). These genes, many of which also share regulation by CREB, collectively enhance synaptic plasticity by promoting spine formation, regulating receptor composition (including AMPA and NMDA receptors), and even facilitating synaptic adhesion (Schmeisser et al., 2012). In essence, NF-κB functions as a molecular hub that translates transient synaptic signals into durable changes in synaptic structure and function, thereby supporting the maintenance of long-lasting memory.

Additionally, NF-κB intersects with BDNF signaling, a critical pathway for synaptic plasticity, neuronal survival, and cognitive function. In AD, disruptions in BDNF/NF-κB crosstalk can compromise neurotrophic support and exacerbate amyloid-β and tau pathology (Gao et al., 2022). NF-κB activation suppresses BDNF expression, while BDNF deficiency may impair neuronal resilience to inflammatory insults, creating a vicious cycle of neurodegeneration (Jones and Kounatidis, 2017). Moreover, chronic activation of NF-κB in the hippocampus and entorhinal cortex has been shown to impair neurogenesis and synaptic function, reducing the generation and integration of new neurons and contributing to cognitive decline (Mattson and Meffert, 2006). Importantly, pharmacological inhibition of hyperactive NF-κB in animal models restores neuronal excitability and spatial memory, supporting the concept that NF-κB blockade can ameliorate cognitive deficits (Soleimani et al., 2025). Together, these pathways underscore the multifaceted role of NF-κB in mediating neuroinflammation, impaired neurotrophic signaling, and reduced neurogenesis—each of which contributes to the progression and persistence of Alzheimer’s disease.

### 4.3 Immune system and cognitive reserve

Studies have shown that physiological factors such as higher education and cognitive activity can protect against AD (Wada et al., 2018; Sando et al., 2008). Subjects with higher education showed reduced odds ratio of developing AD, the effect reported was dose-dependent, meaning that the protective effect of education increased with the increase of years in education (Sando et al., 2008). Cognitive activity, as in participating in cognitively stimulating tasks such as reading a newspaper, playing puzzles and card games has been demonstrated to reduce cognitive decline due to aging by 47%. Moreover, cognitive activities were also shown to reduce the risk of developing AD by 33% in a four and half year’s study (Wilson et al., 2002). Higher education and cognitive activities also appear to protect against depression (Bjelland et al., 2008); a meta-analysis that included 36 studies varying between cross-sectional and longitudinal studies concluded that elderly with lower education were at higher risk of depression with odds ratio of 1:58 (Chang-Quan et al., 2010). Moreover, individuals with higher premorbid intelligence are thought to have a greater buffer that can help mitigate the cognitive commonly associated with depression (Venezia et al., 2018).

A plausible link between previously mentioned domains is most likely brain connectivity and synaptic enhancement. Specifically, higher education is hypothesized to protect against AD by increasing cognitive reserve, defined as the brain adaptability that might explain the discrepancies in cognitive abilities and daily functions in response to aging, pathology, or insult. In other terms, education may mediate the ability of the brain to compensate against cognitive decline by recruiting alternative neural networks developed through years of education. The molecular mechanisms involved in cognitive reserve are poorly understood. A study utilized MRI-based cortical thickness analysis to identify brain regions associated with higher educational levels. Following this, the researchers explored the transcriptional architecture of these regions by utilizing brain-wide regional gene expression data and GSEA analysis (Bartrés-Faz et al., 2019). The GSEA analysis revealed a strong enrichment of gene sets related to neurotransmission and immune response in years of education-related areas. Notably, genes involved in ionotropic neurotransmission, including those coding for receptors for glutamate, GABA, acetylcholine, and serotonin, were predominantly upregulated. This suggests an enhanced role of excitatory and inhibitory signaling in these areas, possibly contributing to the cognitive processes associated with years of education. The identification of neuropeptide signaling, G-protein-coupled signaling, and purinergic pathways highlight the complexity of neurotransmission regulation in these regions. Additionally, the immune response gene sets were notably enriched, featuring genes involved in pathogen recognition (TLR3 and 7), cytokine signaling, and immune activation (TGFB1, AIF1, PYCARD). Findings from literature support the role of TLR7 in memory formation and cognition, activating TLR7 can enhance contextual fear memory, suggesting that TLR7 signaling directly influences memory consolidation (Kubo et al., 2012). Another study shows that TLR7 deletion alters the expression profiles of genes related to neural function particularly synaptic proteins related to neuronal development and differentiation such as GRW2A, DAG67, and GRM5 (Hung et al., 2018a). Recent findings also underscore the critical role of TLR9 in memory formation (Jovasevic et al., 2024). In this study, hippocampal neurons were shown to experience double-stranded DNA breaks following learning, which in turn activated TLR9 signaling. This receptor-mediated cascade engaged several essential processes—DNA repair, centrosome function, and the formation of perineuronal nets—that are crucial for stabilizing and consolidating memory traces. Importantly, disruption of TLR9 function resulted in impaired memory and increased genomic instability, emphasizing the pathway’s significance in maintaining cognitive function. The enrichment of TLR3 and TLR7 in education-related brain regions suggests that, similar to TLR9, they may contribute to memory formation and the establishment of cognitive reserve. It is important to note, however, that these receptors differ in their signaling pathways and brain region specificity. TLR3 generally signals via the TRIF pathway (Hung et al., 2018b), whereas TLR7 predominantly utilizes MyD88 (Fishelevich et al., 2011). Moreover, studies such as Jovasevic et al. have focused on TLR9 in the hippocampus (Jovasevic et al., 2024)—a key area for memory formation—the current findings relate to cortical regions. This suggests that while TLR3 and TLR7 might share a convergent role in supporting cognitive functions, their specific contributions could vary depending on the brain area and the receptor-specific signaling cascades involved. This indicates that immune processes might not only play a protective role but could also influence synaptic functioning and plasticity in these brain areas.

The involvement of immune response in cognitive reserve is further supported by findings from Yegla and Foster (2022), in their study, the researchers identify immune-related pathways as significant contributors to cognitive resilience. Specifically, the study highlights genes such as TREM2, C1qa, and P2RX7 that play crucial roles in microglial activation and neuroinflammation. These genes were found to be enriched in cognitive reserve-related regions, emphasizing the potential link between immune function and cognitive resilience. The overlap in genetic regulation between cognitive reserve and AD suggests that the immune mechanisms contributing to cognitive resilience may also influence AD pathology. These findings imply that individuals with a genetic profile favoring a robust, well-regulated immune response could potentially counteract or delay the detrimental effects of AD-related neuroinflammation. In essence, the same regulatory pathways that bolster cognitive reserve might provide neuroprotection, offering promising targets for therapeutic interventions that not only enhance cognitive resilience but also mitigate the progression of AD.

Environment enrichment (EE) studies in rodents may closely model cognitive reserve (Nithianantharajah and Hannan, 2009), the concept of EE is allowing rodents to have enriched sensory and cognitive and motor stimulation compared to normal housing conditions. Many EE studies have shown positive physical and mental effects on rodents, including enhanced cognitive function and reduced depressive symptom (Brenes et al., 2020; Huang et al., 2021; Cordner et al., 2021; Wei et al., 2020). EE was found to delay cognitive impairments in AD mouse model, AD mice treated with EE showed increased synaptic density and reduced neural inflammation.

Interestingly, serum levels of IFN-*γ* were elevated in EE treated mice which in turn enhanced the secretion of exosomal micro-RNA 146a (miR-146a) from choroid plexus cells and possibly mediated the cognitive rescue (Nakano et al., 2020). miR-146a acts as a negative feedback regulator of astrocytes inflammation by inhabiting NF-κB (Iyer et al., 2012). Similarly, EE was found to enhance cognitive function examined by spatial learning via the Morris water maze, those mice were found to have more complex dentate gyrus of the hippocampus. This enhancement was accompanied by an increase in protein levels of brain derived neurotrophic factor (BDNF), phosphorylated cyclic adenosine monophosphate (cAMP) response element-binding protein (pCREB), both reported to be important regulators of hippocampal neurogenesis and neuroplasticity. CREB is a transcription factor that induces and regulates many genes related to neurotransmitters, growth and transcriptional factors, and metabolic enzymes. CREB involvement in neural plasticity and memory is vastly proven by studies on CREB knockout mice that presented cognitive dysfunctions shown in fear conditioning tests. Moreover, mice that constitutively express CREB were reported to have a lower threshold for induction of late phase long term potentiation (Sakamoto et al., 2011).

### 4.4 Alzheimer’s as an autoimmune disease

Recent evidence has introduced the provocative hypothesis that AD may, in part, be driven by autoimmune processes. Traditionally, chronic neuroinflammation has been recognized as a feature of AD; however, emerging data suggest that dysregulation in the brain’s immune surveillance may lead to an autoimmune reaction against neuronal components. This autoimmunity could contribute to the progressive synaptic loss and neurodegeneration characteristic of AD. The proposed autoimmune aspect of AD raises intriguing questions about the underlying mechanisms, suggesting that it might arise from, or be exacerbated by, maladaptive immune responses—albeit at different points along a spectrum of immune dysfunction.

Genetic studies have revealed robust associations between AD risk and polymorphisms in major histocompatibility complex (MHC) genes, including the human leukocyte antigen (HLA) system (Kunkle et al., 2019; Marioni et al., 2018; Misra et al., 2018; Jones et al., 2015), which are well recognized for their roles in autoimmune disorders such as multiple sclerosis and systemic lupus erythematosus (Alcina et al., 2012; Furukawa et al., 2014). In addition, large-scale genomic analyses have identified overlapping risk variants in genes like CLU, CR1, and BIN1 that connect AD pathology with autoimmune-mediated processes (Yokoyama et al., 2016; Hao et al., 2019; Gagliano et al., 2016). Compounding this genetic predisposition, disruptions in the blood–brain barrier—a hallmark and risk factor of AD—allow immune cells and autoantibodies to infiltrate the central nervous system, triggering pathological immune responses such as antibody-dependent complement activation that promote amyloid-β plaque formation and microglia-induced neuronal death (Sweeney et al., 2018; D'Andrea, 2003).

In a recently suggested novel model, the AD^2^ model reconceptualizes AD as a condition driven by dysregulated innate immunity and autoimmune-like responses rather than solely by protein aggregation (Weaver and Donald Weaver, 2023). In this framework, diverse stimuli—including infection, trauma, ischemia, pollution, metabolic syndrome, and notably, depression—act as triggers that release damage- or pathogen-associated molecular patterns (DAMPs/PAMPs). These molecular signals are sensed by innate immune receptors, initiating an inflammatory cascade. Key early events include the activation of the NLRP3 inflammasome and other pattern recognition receptors, which together foster the release of proinflammatory cytokines. Concurrently, amyloid-β, traditionally viewed as merely a pathological aggregate, is reinterpreted as an immunopeptide with antimicrobial properties. Under normal circumstances, amyloid-*β* serves protective roles; however, when excessively produced or misdirected by persistent immune activation, it can aggregate and further stimulate innate immune responses, perpetuating neuroinflammation.

Depression is highlighted as a possible trigger within this cascade (Weaver and Donald Weaver, 2023). Chronic depressive states are associated with prolonged stress and systemic inflammation (Kim et al., 2022), which may prime the innate immune system, enhance NLRP3 activation, and promote amyloid-β deposition. Together, these events create a self-perpetuating cycle where sustained immune activation leads to synaptic dysfunction, neuronal loss, and ultimately, the cognitive decline seen in Alzheimer’s disease.

In another attempt to frame the autoimmune hypothesis for AD, Arshavsky (2020) proposed that the innate immune response is directed toward specific proteins that are specific for engram neurons. In his explanation, Arshavsky hypothesized that creating memories might be similar to stem cell differentiation, in which epigenetic mechanisms are involved in coding long-term memories within the DNA. As such, these modifications are accompanied by protein synthesis that is unique to the engram neurons. Consequently, the adaptive immune system may identify these proteins as non-self and trigger an autoimmune response toward the engram neurons.

The emerging view of Alzheimer’s disease as an innate autoimmune condition adds a critical layer to our understanding of its pathophysiology, one that bridges immune dysregulation with profound alterations in brain metabolism. Chronic activation of the immune system, whether through microglial priming, complement cascade engagement, or sustained autoantibody responses, imposes a persistent metabolic burden on the brain Immune cells (Pathak et al., 2024), particularly microglia and astrocytes, shift toward pro-inflammatory phenotypes that rely on glycolytic metabolism, generating ROS and pro-inflammatory cytokines (Orihuela et al., 2016; Liddelow et al., 2017). Meanwhile, neurons exposed to this inflammatory milieu experience mitochondrial dysfunction, oxidative stress, and impaired nutrient utilization, hallmarks of metabolic failure observed in AD (Isaac, 2016; Swerdlow, 2017).

## 5 Brain metabolism in AD

Compared to its size, the brain consumes large amounts of energy that are essential for its survival and function. Glucose is the main source of energy in the brain, and unlike other tissues in the body, the brain lacks a reserve of energy source, which renders the neurons vulnerable to glucose fluctuations, this along with the complex architecture of the neurons necessitates the need for a cellular control systems and molecular pathways that closely monitors energy availability and expenditure. Different cells in the brain rely on different energy production pathways, under physiological conditions neurons and microglia predominantly undergo oxidative phosphorylation (OXPHOS), where a series of steps fully oxidize glucose and its metabolite pyruvate to produce the end product as ATP. Astrocytes depend on glycolysis to produce energy from pyruvate, and under hypoxic conditions, can produce lactate through aerobic glycolysis. However, these cells undergo metabolic reprogramming under offensive conditions such as infection, stress, or starvation. The metabolic profile of the brain in AD had gained a lot of attention as a key component of both the initiation and progression of the disease (Muñoz-Torrero et al., 2022; Głombik et al., 2020). In the following section of this review, the focus will be on the changes in energy production in neurons, astrocytes and microglia in AD.

### 5.1 Neuron metabolic changes

Neurons are highly susceptible to mitochondrial dysfunction due to their inherent characteristics. Axonal mitochondria are crucial for impulse conduction, and the length of the axon requires varying ATP levels along its length. This is managed by distributing mitochondria through multiple microtubules and neurofilaments. Ion channels and energy-dependent pumps, like sodium and potassium ATP pumps, are vital for axonal excitability and restoring baseline conditions, so any disruption in energy availability can significantly impair neuronal function. Neurons rely on glucose from the blood, regulated by the BBB and GLUT. Once glucose crosses the BBB, it serves not only as an energy source but also as a precursor for synthesizing fatty acids, other lipids, amino acids for neurotransmitters, and sugars for nucleotides.

As mentioned earlier, signs of disrupted glucose metabolism are seen early in the disease, and even decades before any clinical signs (Kumar et al., 2022). Reduced brain glucose can impair memory and synaptic plasticity as well as increase tau phosphorylation (Lauretti et al., 2017). Samples from the dorsolateral prefrontal cortex showed a wide profile of dysregulated metabolic features including bioenergetics, and neurotransmitters by-products, noting that the metabolic dysregulations seen were driven mostly by tau pathology rather than Aβ (Batra et al., 2023).

Recently, a study on postmortem AD brains found that impaired glucose phosphorylation and increased levels of several tricarboxylic acid (TCA) metabolites, except for citrate, were observed. The extent of the phosphorylation impairment and the rise in TCA cycle metabolite levels were negatively and positively correlated, respectively, with the clinical symptoms of AD (Kurano et al., 2024).

In neurons, OXPHOS is associated with increase in the production of reactive oxygen species and free radicals and results in oxidative damage, specifically to the mitochondrial membrane leading to increase in electron leak and to key enzymes in mitochondrial bioenergetics such as pyruvate dehydrogenase and *α*-ketoglutarate. The effect on such enzymes includes a reduction in their activity and the subsequent attenuation of the electron transport chain (ETC) efficiency. Neuronal progenitor cells derived from LOAD patients showed alterations in energy production between mitochondrial respiration and glycolysis, resulting from changes in the processing of bioenergetic substrates and the transfer of reducing agents. This included reduced levels of NAD/NADH, decreased glucose uptake, and lower response rates to insulin/IGF-1 signaling. Additionally, there was a reduction in insulin receptor and GLUT-1 densities, along with changes in the metabolic transcriptome (Ryu et al., 2021).

A study on rat AD model that underwent glucose administration after overnight fasting showed that glucose uptake in the entorhinal cortex and hippocampus was reduced, indicating a possible decrease in GLUTs in these areas (Joo et al., 2022). Indeed, the study on the autopsy program of the Baltimore Longitudinal Study of Aging showed an increase in brain glucose levels, increase in glycolytic flux and lower levels of GLUT-3 in AD brains, all of which correlated with the disease severity and hallmarks of AD pathology (An et al., 2018). Glucose-6-phosphate dehydrogenase (G6PD)is essential for the maintenance of the nicotinamide adenine dinucleotide phosphate (NADPH) pool, it acts as an oxidoreductase in the pentose phosphate pathway (PPP), a key metabolic pathway for glucose metabolism (Tiwari, 2017), and was found to be reduced in AD (Ulusu, 2015). Interestingly, G6PD overexpression in AD mice model attenuates the cognitive impairment, enhanced physical abilities, and reduced the stress load in the brain cortex (Correas et al., 2024). A recent study that investigated cerebral glycolytic metabolism in 5XFAD mice using molecular imaging found that the AD model exhibited age-related alterations in glucose uptake compared to wild type. These changes likely reflect the progressive metabolic dysfunction associated with AD pathology. The Analysis also revealed that the structure of metabolic covariance networks varied significantly with age and sex. In 5XFAD mice, there was a notable disruption in metabolic coupling, indicating that the coordination of metabolic activity among different brain regions deteriorates as AD progresses (Chumin et al., 2024). Moreover, a pilot metabolomics study on postmortem pre-frontal cortical tissue of AD patients found that energy metabolites derived from glucose in the glycolytic and PPP, as well as the ketone body β-hydroxybutyrate, were consistently reduced in the AD brain compared to the control brain (Patel et al., 2024).

### 5.2 Astrocytes metabolic changes

Astrocytes play essential roles in the normal functioning of nervous tissue. They express numerous receptors that enable them to sense neuronal activity and clear neurotransmitters such as glutamate, ATP, GABA, adenosine, and endocannabinoids. This helps maintain synaptic transmission, prevent excitotoxicity, and provide neuroprotection. Additionally, astrocytes promote the formation of synapses by producing and secreting vital factors like cholesterol, glypicans, and thrombospondins (Sidoryk-Wegrzynowicz et al., 2010).

In AD, the role of astrocytes is widely acknowledged (Garwood et al., 2017; González-Reyes et al., 2017). APOE, a significant genetic risk factor for LOAD, is predominantly expressed in astrocytes in a healthy brain (Yu et al., 2014). In the presence of senile plaques, astrocytes become reactive, showing morphological hypertrophy with thicker processes and elevated levels of intermediate filament proteins like glial fibrillary acidic protein (GFAP), vimentin, nestin, and synemin (Escartin et al., 2021). In AD, there is often a severe disruption or complete loss of interlaminar astrocytes. Additionally, astrocytes may be involved in β-amyloid production by upregulating BACE1 and the APP in the diseased brain (Frost and Li, 2017).

Astrocytes, the primary regulators of energy in the brain, play a crucial role in maintaining brain homeostasis. They restore ion gradients, such as those involved in postsynaptic and action potentials, and manage the uptake and recycling of neurotransmitters, all of which require significant brain energy. Astrocytes are situated between blood vessels and neurons, placing them in an ideal position to regulate glucose flow into the brain and act as metabolic sensors.

Glutamate, the primary excitatory neurotransmitter in the brain, is critical for synaptic plasticity and neuronal health. Since glutamate cannot cross the BBB, it relies on glucose to act as a precursor for its synthesis in astrocytes. Under normal conditions and following transmission, glutamate is removed by astrocytes following sodium gradient and through glutamate transporters (GLAST (EAAT1), GLT1 (EAAT2)). Subsequently, glutamate is either transformed into glutamine or utilized as cellular fuel in cases of high concentrations.

It has been suggested that metabolic changes in astrocytes are triggering factors in the AD pathology, as they result in reduced Aβ clearance and mediate the increase in its production. Moreover, astrocyte metabolic dysfunction produces oxidative stress and neuroinflammation that are central to the disease progression in that it facilitates the hyperphosphorylation of tau and the formation of NFTs (Cai et al., 2017; Yan et al., 2013). Human iPSCs- derived astrocytes and neurons carrying familial AD mutations (*PSEN1* or *APP*) showed higher levels of glycolysis and oxidative glucose metabolism along with elevated levels of glutamate synthesis. Moreover, astrocytes showed decreased expression of EAAT2, which reduced glutamate reuptake (Salcedo et al., 2024). The increase in glutamate, though may offer an early oxidative protection through maintaining glutathione content (an important antioxidant in the brain), however activated astrocytes surrounding Aβ plaques are correlated with disease progression, having a direct effect on synaptic plasticity and neurotransmission (Verkhratsky et al., 2010).

In 5XFAD mice, astrocytes showed reduced TCA cycle activity and reduced glutamine synthesis early in the disease, this attenuated neuronal GABA synthesis in the hippocampus (Andersen et al., 2021). Single-cell transcriptional profiling of astrocytes in prefrontal cortex of AD patients revealed a metabolic reprogramming characterized by reduced glycolysis and TCA, and reduced glutamate metabolism, interestingly, glutamine synthetase (GLUL) and glutamate dehydrogenase (*GLUD1*) expression were downregulated, these enzymes regulate key glutamate metabolism fluxes (Fan et al., 2023) and were shown to be an indicator of memory decline in AD patients (Neuner et al., 2017).

Magnetic resonance spectroscopy in AD patients showed increased levels of Myo-inositol and lactate levels, indicating a relation between activated astrocytes and altered energy metabolism in the brain, these metabolites were positively associated with clinical signs of cognitive dysfunction (Hirata et al., 2024). Interestingly, My-inositol was reported as a marker for MCI in mice, the rise in its levels was accompanied by increase in GFAP, an intermediate filament protein that makes up the framework of astrocytes cytoskeleton, and a maker of astrocytic activity (Ebert et al., 2021). Similarly in AD patients, plasma levels of GFAP correlated with Myo-inositol levels only in carriers of the *APOE4* allele (Spotorno et al., 2022). Recently, it was found that the earliest increase in CSF Aβ_42/40_ triggered an increase in plasma GFAP, which partially explained the association between CSF Aβ and brain Aβ accumulation measured by PET (Pelkmans et al., 2024). These findings point toward a key involvement of astrocytic activity, changes in metabolic profile and the early imbalance between soluble, and aggregated Aβ.

### 5.3 Microglia metabolic changes

Microglia are the innate immune cells of the CNS, they comprise 10–15% of glial cells in the brain. Microglia can be found in either activated (pro-inflammatory, producing cytokines such as IL-1 β INF- *γ*, IL-8, TNF-*α* and IL-6) or dormient state (anti-inflammatory, producing IL-10 and IL-4). Besides their defense against pathogens, microglial processes enable them to survey the microenvironment in the brain, communications between microglia and neurons enables microglia to survey and maintain efficient neural activity by pruning old or defective neural connections (Wolf et al., 2017).

The high energy consumption of microglia is generally met through glucose metabolism. Microglia have the ability to switch between glycolysis and OXPHOS based on energy demand. In resting state, microglia produce ATP through OXPHOS, however under inflammation or stress conditions, microglia switch to anaerobic glycolysis for faster energy production (Aldana, 2019). To compensate for the energy deficiency, microglia enhance glucose absorption through GLUT-1 receptors. Studies have shown that inhibition of OXPHOS or ETC in cultured microglia/macrophages activates microglia and causes morphological changes and activation of molecular pathways such as mitogen-activated protein kinase (MAPK)/NF-κB pathway and NOD-like receptor protein 3 (NLRP3) inflammasomes, Increased production of pro-inflammatory cytokines and the rapid buildup of damaged mitochondria result in the dysfunction and apoptosis of microglia and macrophages (Li et al., 2022).

Reduced nutrient availability, including glucose, and altered mitochondrial ETC activity in Alzheimer’s disease impair energy supply, suggesting oxidative stress damage and metabolic reprogramming in microglia. It was also reported that cultured microglia chronically exposed to Aβ lead to metabolic reprogramming and entering a state of tolerance which can be reversed by INF-*γ* (Baik et al., 2019). This reprogramming was dependent on the mTOR-HIF-1α pathway. Interestingly, microglia isolated from 5XFAD mice and treated with Aβ showed differentially enriched biological processes related to metabolic process and pathways including carbohydrate derivative metabolic process, primary metabolic process, and carbon metabolism, indicating a clear metabolic dysregulation (Baik et al., 2019).

Moreover, activated microglia shows an increase in succinate levels caused by a break in the Krebs cycle at succinate dehydrogenase (SDH) (Mills et al., 2016). Succinate is crucial in oxidative metabolism. As an intermediate in the TCA cycle, it directly interacts with the ETC, providing an alternative way for ATP production through oxidative metabolism (Giorgi-Coll et al., 2017). Succinate is a known proinflammatory metabolite that increases during macrophages activation. LPS-activated microglia showed high glycolysis and mitochondrial respiration levels, the metabolic rewiring was reversed by inhibiting SDH, causing the cultured microglia to shift back to M2 phenotype (Sangineto et al., 2023).

**Figure 1:**
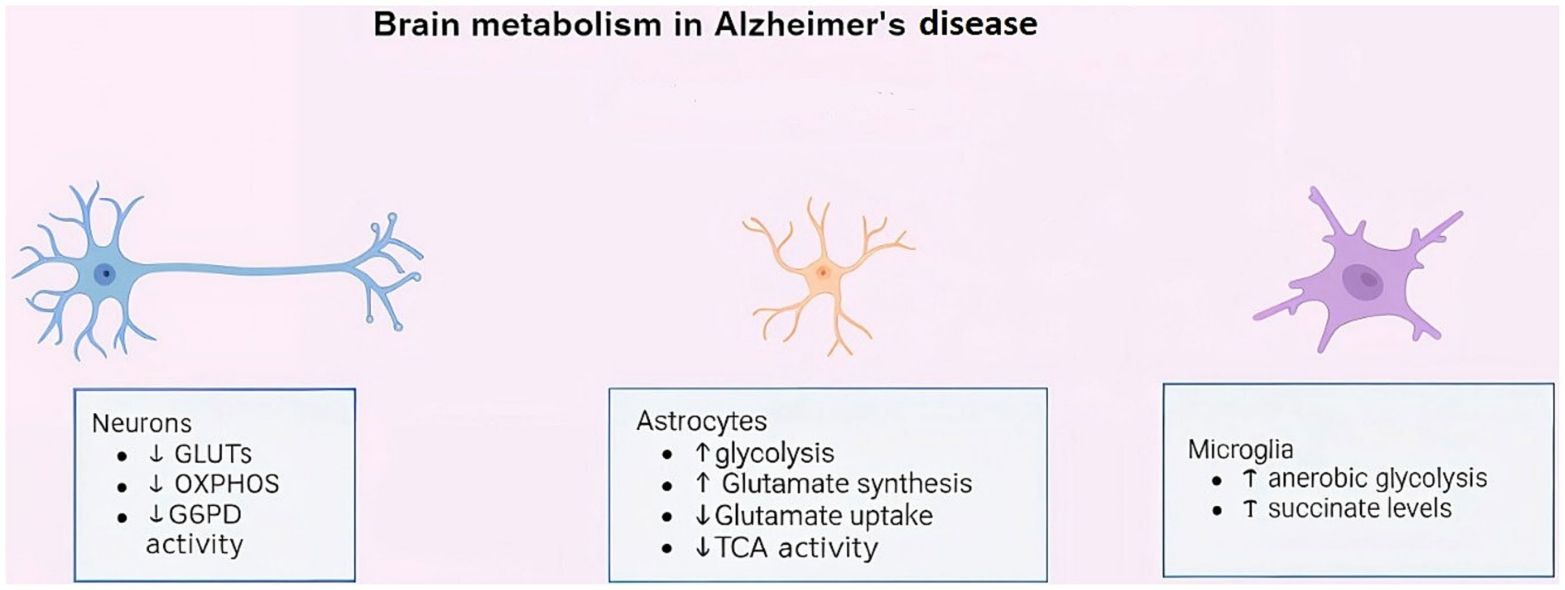
Schematic illustration of metabolic changes in neurons, astrocytes, and microglia in. Key associated genes include SLC2A1, G6PD in neuronal glucose and OXPHOS dysregulation in AD; GLUL, SLC1A2, LDHA in astrocytic glutamate and glycolytic pathways; and NFκB, NLRP3, GLUT1, GLUT3 in microglial metabolic reprogramming.

Alterations in the metabolic profiles of neurons, astrocytes, and microglia (Figure 1) play a critical role in the pathophysiology of AD. These cellular changes disrupt normal brain homeostasis and contribute to neurodegeneration, inflammation, and cognitive decline observed in this disorder.

## 6 Autophagy in AD

Autophagy, the cellular process responsible for the lysosomal degradation and recycling of cellular components (Cao et al., 2021), and is intimately intertwined with the pathogenesis of AD (Zhang et al., 2021). In autophagy, a double membraned vesicle segregates damaged organelles and toxic protein accumulation. Dysregulation of autophagy has emerged as a key contributor to the accumulation of misfolded proteins, oxidative stress, and neuroinflammation, all hallmark features of AD (Zhang et al., 2021). Three types of autophagy are recognized, microautophagy, chaperone mediated autophagy, and macroautophagy. Categorization of autophagy depend on the how the carried cargo Is delivered to lysosomes. Macroautophagy is the major type (hereafter referred to as autophagy). Autophagy starts with a double membraned precursor that surrounds and engulfs cytoplasmic components, this precursor then elongates until it forms a double membrane vesicle (autophagosome) that is transported by microtubules to fuse with lysosome (autolysosome), ultimately leading to degradation of the autophagosome contents (Cao et al., 2021).

Autophagy machinery requires different proteins specifically autophagy-related genes (ATG) and is carefully controlled by various signaling pathways such as the inhibition of mTOR complex by adenosine monophosphate-activated protein kinase (AMPK), which facilitates the activation of ULK1 kinase complex and the production of PI3P lipids which are essential for the recruitment of ATG proteins (Condon and Sabatini, 2019). The induction of autophagy triggered by high AMP/ATP ratio is critical for neuronal homeostasis and survival, as post-mitotic neurons do not have the ability to dilute accumulated proteins through cell division.

**Figure 2:**
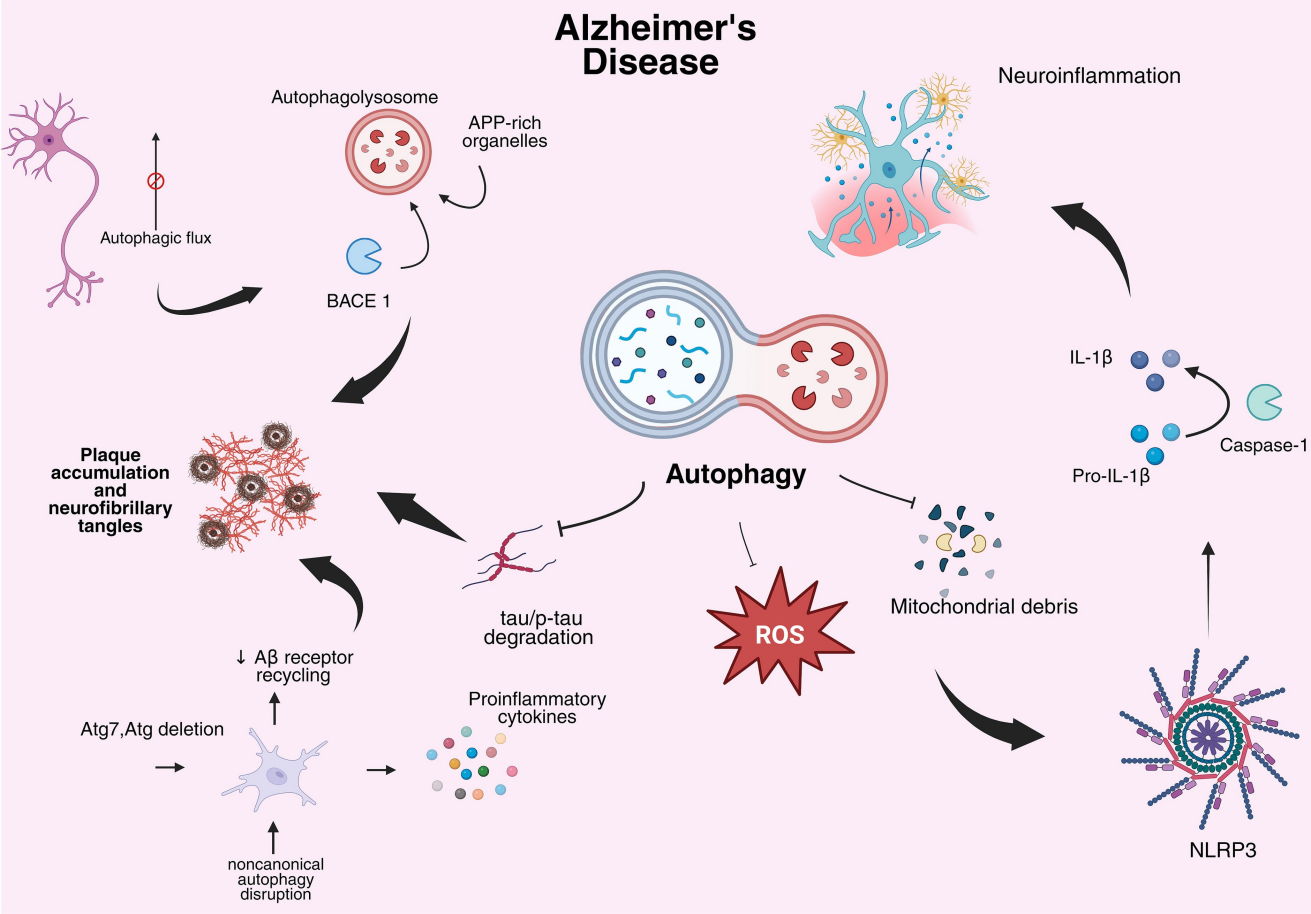
Autophagy modulation in AD. Dysregulation of autophagy genes (BECN1, ATG5, BACE1) leads to APP accumulation and impaired tau degradation (GSKβ3). Impaired mitophagy and ROS production involve NLRP3, CASP1, and IL1B, contributing to neuroinflammation. The figure summarizes mechanisms supported by gene- and protein-level evidence.

In AD, autophagy dysregulation is believed to be highly implicated in the accumulation of Aβ senile plaques and other major hallmarks such as tauopathies and neuroinflammation (Figure 2) (Uddin et al., 2018). The cleavage of APP takes place in autophagosomes of APP-rich organelles, which is then degraded by lysosomes, this is particularly important because in AD, the maturation of autophagolysosomes (autophagosomes that had merged with lysosomes) and their subsequent migration toward the neuronal body is hindered. This leads to accumulation of autophagic vacuoles that favors the A*β* accumulation (Nixon, 2007). Moreover, mature BACE1 is internalized into endosomes where the acidic media is ideal for its activity, then, there is where it is eventually degraded by lysosomes (Huse et al., 2000; Yang et al., 2003). Because of the disrupted migration of autophagosomes to the soma in AD brain, BACE1 is accumulated augmenting APP cleavage in axons and exasperating AD pathological aspects (Feng et al., 2017).

Following APP cleavage the APP-β secretase cleaved C-terminal fragment substantially activates Rab5- Rab5 is a small GTPase that plays a crucial role in the regulation of endocytosis and endosomal trafficking within cells- which causes an early neuronal endosomal dysfunction eventually leading to dysfunctional autophagy, this effect maybe mediated by can hindering the proper delivery of autophagic cargo to lysosomes, or downregulation of the Akt signaling (Pensalfini et al., 2020). It was also reported that overactivated Rab5 also results in hyperphosphorylated tau through the activation of GSK3β (Pensalfini et al., 2020).

Recently, it has been demonstrated that treating rats with AlCl3-induced AD with mesenchymal stem cell-derived exosomes reduces cognitive impairments by upregulating autophagy genes and downregulating mTOR genes and its target genes. This was accompanied by a decrease in APP and its metabolite and Aβ oligomers, which could be due to controlling APP processing through inducing autophagy (Ebrahim et al., 2024).

Tau protein can be degraded by the autophagy-lysosome pathway, more so when the ubiquitin-proteasome system is inhibited, it was shown that proteasomal inhibitors favored the clearance of tau protein through autophagy as apparent by the increase in LC3-II (a protein that is essential for processing of cellular components in the forming phagophore) and increased number of autophagosomes in rat primary neurons (Hamano et al., 2021). Interestingly, a dose-dependent reduction in tau and p-tau was seen when administrating mTOR inhibitors in an *in vitro* model of tauopathy, this reduction was associated with upregulation of autophagy-lysosomal pathway markers such as LC3-II and ATG12/ATG5 indicating autophagosome formation. Moreover, there was an increase in levels of p-p62, which-upon phosphorylation- interacts with LC3-II and transports cargo into the autophagosome (Silva et al., 2020).

Recently, microglial autophagy has gained attention, microglia can contribute to autophagy through phagocytosis/endocytosis. Impairment of autophagy in microglia can lead to accumulation of aggregated proteins and damaged organelles. In AD, autophagy is relevant through two distinct pathways, influencing the inflammatory state of microglia and, increase of autophagy protein-mediated phagocytosis/endocytosis (Plaza-Zabala et al., 2017).

Microglia-specific deletion of *Atg7*, an essential protein in the autophagy process, lead microglia to transition to pro-inflammatory phenotype, along with increase in the gene expression of proinflammatory cytokines, indicating that *Atg7* may control the inflammatory status of microglia *in vitro* (Xu et al., 2021).

Moreover, 9 days of intraperitoneal administration of IFN-*γ* enhanced autophagy induction indicated by increased levels of autophagy markers such as LAMP1, p62, Atg5, and LC3II/I in microglia of APP/PS1 mice, an effect that was probably mediated by suppressing the Akt/mTOR pathway. IFN-γ also protected BV2 cells from Aβ toxicity through upregulation of Atg7 and Atg5 where the use of small interfering RNA to knock down Atg5 expression diminished the protective effect of IFN-γ (He et al., 2020). These results emphasize the important role of autophagy in the protective role of IFN-γ.

Also, inducing microglial autophagy through the activation of peroxisome proliferator-activated receptor-*α* (PPARA) using gemfibrozil and Wy14643 has been linked to the improvement of AD-like symptoms in the APP-PSEN1 mouse model (Luo et al., 2020).

Microglia also engulf apoptotic cells during degeneration, the signaling pathways involved in this process are not fully understood, however the classical complement system works as “tags” to which the microglia identify and respond to. C1q and c3 are frequently reported to act as “eat me” signals to the microglia via the complement receptor CR3 which is exclusively expressed on microglia (Baik et al., 2019; Datta et al., 2020). Following internalization of apoptotic cells via scavenger receptors, the formed phagosome would fuse with the lysosome eventually degrading the target (Li et al., 2021).

Microglial autophagy is essential for recovery from neuroinflammation, in a murine model of multiple sclerosis, it was found that specific deletion of microglial *Atg7* facilitated the accumulation of phagocytosed myelin and exacerbated the disease progression (Berglund et al., 2020).

LC3-associated endocytosis (LANDO), a noncanonical form of autophagy, was found to be an important regulator of microglial inflammatory response and vital for aggregate removal, mice lacking LANDO showed increased levels of pro-inflammatory cytokines and increased neurotoxic A*β*. Remarkably, this effect maybe mediated by LANDOs ability to recycle Aβ receptors and prevent their permanent internalization and degradation (Heckmann et al., 2019).

## 7 Discussion

Over the past two decades, therapeutic development in Alzheimer’s disease has largely centered on targeting amyloid-β aggregation and, more recently, tau pathology (Vaz and Silvestre, 2020; Passeri et al., 2022). Despite advances in biomarker detection and monoclonal antibody development, clinical benefits have remained modest and inconsistent (Vaz and Silvestre, 2020; Passeri et al., 2022), raising questions about whether protein aggregation represents the primary driver of disease rather than a downstream manifestation of broader dysregulation.

Large clinicopathological studies have shown that amyloid burden does not consistently correlate with the severity of cognitive impairment (Nelson et al., 2012; Jack et al., 2018). Moreover, transcriptomic and proteomic analyses of human cohorts, demonstrate that immune and metabolic pathways are often more strongly enriched early in disease stages (De Jager et al., 2018; Mathys et al., 2019). These findings suggest that aggregation-centric approaches may overlook upstream regulatory processes that shape the disease course.

When genetic, transcriptomic, and proteomic layers are examined together, immune-related pathways repeatedly emerge as enriched across datasets. Genome-wide association studies consistently identify AD risk loci enriched for immune-regulatory genes, including TREM2, CD33, CR1, and the APOE locus (Kunkle et al., 2019; Bellenguez et al., 2022). Single-cell transcriptomic analyses of early AD brains demonstrate upregulation of complement components, APOE-associated inflammatory programs, and microglial activation signatures (Mathys et al., 2019; Olah et al., 2020). Multi-omics analyses consistently identify immune and glial-associated modules as significantly enriched and associated with AD pathology and cognitive decline, indicating that immune regulation constitutes a major molecular dimension of the disease (Wingo et al., 2021; Johnson et al., 2020).

Importantly, stress-responsive and glucocorticoid-regulated pathways intersect with these immune networks. Notably, glucocorticoid signaling intersects with transcriptional and proteomic immune modules identified in multi-omics network analyses, suggesting that stress-related endocrine signals may act as upstream modulators of disease-associated molecular programs. Chronic stress and HPA-axis dysregulation have been linked to hippocampal vulnerability, neuroinflammation, and accelerated cognitive decline (Sotiropoulos et al., 2011; Lupien et al., 2009). Elevated cortisol levels in prodromal stages predict disease progression (Popp et al., 2015). Taken together, multi-layer analyses suggest that immune regulation, particularly under stress-related conditions, represents a recurring biological axis in AD.

Accumulating evidence challenges the traditional view that cognition is governed exclusively by neuronal networks. Microglia and other immune cells contribute to synaptic pruning, plasticity regulation, and learning-dependent remodeling (Paolicelli et al., 2011; Parkhurst et al., 2013).

In AD, dysregulated microglial responses influence synaptic loss and circuit dysfunction (Hong et al., 2016). Complement-mediated synapse elimination has been implicated in early cognitive decline (Stevens et al., 2007). These findings expand the conceptual framework of cognitive reserve to include immune-mediated synaptic regulation. Cognitive resilience may therefore depend on controlled immune surveillance alongside neuronal integrity.

The hypothesis that AD may involve autoimmune-like components has gained attention. Autoantibodies against neuronal and synaptic proteins have been reported in subsets of patients (Hansen et al., 2017; Doss et al., 2014). While the autoimmune classification remains debated, immune dysregulation clearly plays a mechanistic role in synaptic and cognitive deterioration.

Environmental stressors, infections, and chronic systemic inflammation activate the HPA axis and peripheral immune signaling. Sustained glucocorticoid exposure modulates microglial inflammatory responsiveness (Wohleb et al., 2018; Frank et al., 2007). It is therefore plausible that repeated stress exposure could interact with genetic susceptibility to shift immune balance toward maladaptive states in vulnerable individuals.

This remains a hypothesis rather than a confirmed causal pathway, but it aligns with convergent multi-omics observations.

Recent studies demonstrate that microglial activation is tightly coupled with metabolic remodeling. Inflammatory microglia exhibit shifts toward glycolysis, altered mitochondrial function, and lipid metabolism reprogramming (Orihuela et al., 2016; Baik et al., 2019).

Disease-associated microglia in AD models display altered lipid handling and oxidative metabolism (Nugent et al., 2020; Keren-Shaul et al., 2017). Metabolic state influences cytokine production, phagocytic capacity, and synaptic engagement. Therefore, immune activation in AD cannot be interpreted independently of metabolic regulation. When viewed through a multi-omics lens, genetic susceptibility, stress signaling, immune activation, and metabolic reprogramming appear interconnected rather than isolated domains.

The convergence of genetic risk enrichment, early immune transcriptional activation, proteomic network dysregulation, stress-associated endocrine changes, and metabolic remodeling suggests that AD pathology emerges from coordinated disturbances across regulatory systems. This framework does not negate the role of amyloid or tau but situates them within a broader regulatory context shaped by immune and stress-responsive networks.

Future therapeutic strategies may benefit from targeting regulatory axes that integrate endocrine, immune, and metabolic signals rather than focusing exclusively on aggregated proteins.

## 8 Conclusion

Alzheimer’s disease is increasingly recognized as a multifactorial disorder driven by complex interactions between neuroinflammation, immune dysregulation, metabolic dysfunction, and genetic susceptibility. This notion emphasizes the need for integrating multi-omics data. Genetic studies identify risk loci but are insufficient to explain how those variants are functionally expressed in brain tissue. Transcriptomic analyses reveal early and cell-type–specific changes, yet mRNA levels do not consistently predict protein abundance. Proteomic studies show that many transcriptional signals are buffered, amplified, or redirected at the protein level. Similarly, metabolic alterations reflect functional consequences that cannot be inferred from gene expression alone. The importance here stems from a broad, interconnected view of all levels of the central dogma in the context of AD, without assuming alignment across all layers. Importantly, integrative analyses also reveal heterogeneity among patients, suggesting that immune activation, metabolic adaptation, and proteostatic stress do not occur uniformly across individuals. This variability has direct implications for therapeutic development, as targeting a single pathway without accounting for broader molecular context may limit clinical efficacy Further research should focus on post-transcriptional and post-translational regulatory mechanisms and their upstream modulators to advance diagnostic and therapeutic strategies in Alzheimer’s disease.

## Footnotes

1
https://www.ebi.ac.uk/gwas/home

## Glossary

AD: Alzheimer’s disease
MENA: Middle East and North Africa
Aβ: Amyloid beta
T2DM: type 2 diabetes mellitus
GWAS: genome wide association studies
LOAD: late-onset Alzheimer’s disease
EOAD: early-onset Alzheimer disease
PSEN: presenilin
APP: amyloid precursor protein
GABA: gamma-aminobutyric acid
BACE: beta-site APP-cleaving enzyme
sAPPβ: soluble form of APP beta
APOE: apolipoprotein
LDLR: low-density lipids receptor
VLDLR: very low-density lipids receptor
APOER2: apolipoprotein receptor 2
LRP: LDL-receptor-related protein
GSK3β: glycogen synthase kinase 3β
PKCδ: protein kinase c delta
ABC: ATP-binding cassete
MAP2c: microtubule associated protein 2c
NMDA: N-methyl-D-aspartate
AMPA: *α*-amino-3-hydroxy-5-methyl-4-isoxazolepropionic acid
TR: targeted replacement
EE: environmental enrichment
NF-κB: nuclear factor kappa B
BDNF: brain derived neurotrophic factor
pCREB: phosphorylated cyclic adenosine monophosphate (cAMP) response element-binding protein
LTP: long term potentiation
NDMAR: N-methyl-D-aspartate receptor
Akt: protein kinase B
GLUTs: glucose transporters
IR-S: insulin receptor substrate
PI3K: phosphatidylinositol-3 kinase
mTOR: The mammalian target of rapamycin
AβO: amyloid beta oligomers
BVR-A: biliverdin reductase A
CK1: casein kinase 1
MCI: mild-cognitive impairment
IDE: insulin degrading enzyme
IAPP: islet amyloid polypeptide
HPA-axis: hypothalamic–pituitary–adrenal axis
ACTH: adrenocorticotropic hormone
GR: glucocorticosteroid receptor
LGI: low grade-inflammation
IDO: indoleamine 2, 3-dioxygenase
OXPHOS: oxidative phosphorylation
TCA: tricarboxylic acid
G6PD: Glucose-6-phosphate dehydrogenase
PPP: pentose phosphate pathway
5-HT: serotonin
NE: norepinephrine
SSRI: selective serotonin reuptake inhibitor
CUMS: chronic unpredictable mild stress
GFAP: glial fibrillary acidic protein
GLUL: glutamine synthetase
GLUD1: glutamate dehydrogenase
LDHA: lactate dehydrogenase A
FST: forced swim test
MAPK: mitogen-activated protein kinase
ETC: electron transport chain
SDH: succinate dehydrogenase
ATG: autophagy related genes
PPARA: peroxisome proliferator-activated receptor-alpha
LANDO: LC3-associated endocytosis
CRP: c-reactive protein
NLRP3: NOD-like receptor protein 3
PAMPS: pathogen-associated molecular patterns
ASC: apoptosis associated speck-like protein containing caspase recruitment domain
